# Evaluation of cervical cancer screening program in Gondar city administration public health facilities, Northwest Ethiopia, 2021: mixed method approach

**DOI:** 10.1186/s12885-023-11533-7

**Published:** 2023-10-25

**Authors:** Ketemaw Tsegaye, Asebe Hagos, Hailemichael Kindie, Amare Minyihun, Getachew Teshale

**Affiliations:** 1 University of Gondar, College of Medicine and Health Sciences, Gondar, Ethiopia; 2 Department of Health Systems and Policy, Institute of Public Health, College of Medicine and Health Sciences, University of Gondar, Gondar, Ethiopia

**Keywords:** Evaluation, Visual inspection with acetic acid, Cervical cancer, Gondar, Ethiopia

## Abstract

**Background:**

Cervical cancer is one of the most malignancies in women all over the world. Over 90% of cases occurred in low and middle-income countries with limited resources. Even though cervical cancer is preventable, the Sub-Saharan countries are the most burdened. In Ethiopia 27.19 million women are at risk of acquiring cervical cancer. Although the prevalence of cervical cancer screening among women aged 18 to 69 was around 14%, due to COVID 19 and internal conflict the screening prevalence was lowered to 0.2% by 2022.

**Objective:**

This study aimed to evaluate cervical cancer screening program implementation at Gondar city administration public health facilities, Northwest Ethiopia.

**Methods:**

Single case study design with mixed method evaluation was employed in eight public health facilities of Gondar city administration from March 29 to May 30, 2021. The quantitative data were collected through exit interviews and resources inventory observations. While qualitative data were collected through Key informant interviews, non-participatory observation and document review. A total of 310 clients, 14 key informants, 30 non-participatory observations and six months retrospective document reviews were included in this evaluation. Quantitative data were entered into EPI-data version 4.6 and exported into SPSS version 20 for analysis. For qualitative data; records were transcribed, translated and analyzed in themes. Variables with *P*-value < 0.05 at 95% confidence interval and adjusted odds ratio were used to declare associated variables with client satisfaction.

**Results:**

The overall implementation of cervical cancer screening program with visual inspection with acetic acid was 64.5%. The availability of program resources, compliance of healthcare providers and satisfaction of mothers were 52.3%, 64.3% and 77.1% respectively.

Being educated, having information on cervical cancer screening and the number of lifetime sexual partners were positively associated variables with client satisfaction.

**Conclusion:**

The cervical cancer screening program was judged as partially-implemented and needs urgent improvement based on pre-determined judgment parameters. To implement the program properly and serve more women; human and material resources should be available, providers shall be trained and the health facilities should equip with full infrastructures like electric power supply and separate procedure rooms.

## Background

Cervical cancer is a malignancy arising from the cervix, the lower portion of the uterus. It is the major public health issue around the world with 90% of the cases happening in low-and middle-income resource (LMIC) countries. Approximately 570,000 new cases and 311,000 women deaths were recorded in 2018, [1, 2]. Despite of cervical cancer is preventable, the Sub-Saharan African (SSA) countries are the most burdened which is the second most common cancer-related killer in women [1, 3]. The majority of cervical cancers (more than 80%) in SSA are detected at an advanced stage, which results low survival rates and treatment modalities may be too expensive and inaccessible for many women in low resource countries, including Ethiopia [4]. The World Health Organization (WHO) recommends cervical cancer screening by using human papilloma virus testing wherever possible. Although human papillama virus testing is more sensitive and detects pre-cancers and cancers earlier than cytology, there are currently costs, infrastructure considerations and specificity issues that limit its use in low and middle-income countries. Therefore, WHO accepts the alternative screening approach of visual inspection with acetic acid as part of “screen and treat” programs [5]. But still the screening activities are very low and in many challenges.

In low income countries the screening and treatment of cervical cancer with VIA have many challenges and limitations. A study conducted in countries with low income and high burden of HIV AIDS showed that poorly equipped health facilities and a lack of national cancer prevention policies and programs are the macro level barriers [6]. Another study conducted in Malawi identifies limited infrastructure and lack of trained personnel were the main challenges to implement the screening program [5].

In Ethiopia, one of the developing countries, the cervical cancer burden is high that an estimated 7095 new cases and 4732 women die from the disease annually [7]. Cervical cancer is Ethiopia's second most common malignancy, with 27.19 million women at risk of acquiring the illness. Although the prevalence of cervical cancer screening among women aged 18 to 69 was around 14% [8], due to COVID 19 and internal conflict the screening prevalence was lowered to 0.2%. Regular screening can prevent the illness in around half (45%) of cases in their age of 30 s and three quarters (75%) of cases in their age of 50 s and 60 s [9].

The government of Ethiopia also expands cervical cancer screening by using VIA clinics and recommends treatments to women who are 30–49 years and who are at high risk. However, care usage among eligible and high-risk women is extremely low [10].

Cervical cancer fatalities will be reduced significantly if women between the ages of 30 and 49 are screened at least once in their lives. The majority of high-grade precancerous lesions are discovered between these ages, making this the best age to test women. The screening interval should be every 3–5 years for first screen negative women and not more than 3 years for human immune virus (HIV) positive women [11].

Despite cervical cancer is the most common prevalent cancer case in Ethiopia, early screening and treatment is too low [12]. Therefore, evaluating this program is very important to identify gaps and provide information needed to improve the program.

## Description of cervical cancer screening program

### Program stakeholders

The stakeholders of the program were identified during the evaluability assessment phase. These stakeholders were Federal Ministry of Health (FMoH), Amhara Regional Health Bureau (ARHB), Central Gondar Zone Health Department (CGZHD), Gondar city administration health office, women aged 30–49 and healthcare providers at the selected public health facilities. Those who can physically participated stakeholders were involved with development of evaluation questions, indicators, judgment matrix. The other stakeholders were considered during data collection, result dissemination and utilization of the information (Table 1).

**Table 1:** Stakeholder analysis matrix for evaluation of cervical cancer screening program implementation in Gondar city administration health centers, 2021

Stakeholders	Role in the program	Role in the evaluation	Way of communication
FMoH	-Coordinate the program implementation at national level -Policy formulation -budget allocation	-finding user -facilitate means to disseminate evaluation findings through conference	Phone Email
ARHB	-provide training to HCWs -supportive supervision -material and budget support	-finding user -facilitate means to disseminate evaluation findings through conference	Phone Email
CGZHD	-Coordinating campaign for community awareness -Facilitate and coordination the program with funding, trainings, monitoring and evaluation	-Provides evaluation data, -finding users -indicator, evaluation questions and judgment development	Face to face -phone -written -email
Gondar town administration health office	-program implementer -Facilitate and coordination the program with funding, trainings, monitoring and evaluation	-Provides evaluation data -finding users -indicator, evaluation questions and judgment development	-Face to face -Phone -Formal letter
Health care providers	- implementer (perform screening and management)	- Provides evaluation data -indicator, evaluation questions and judgment	-Face to face
30–49 years women	-service users	-provide evaluation data -benefited from findings	Face to face

### Program logic model

**Fig. 1:**
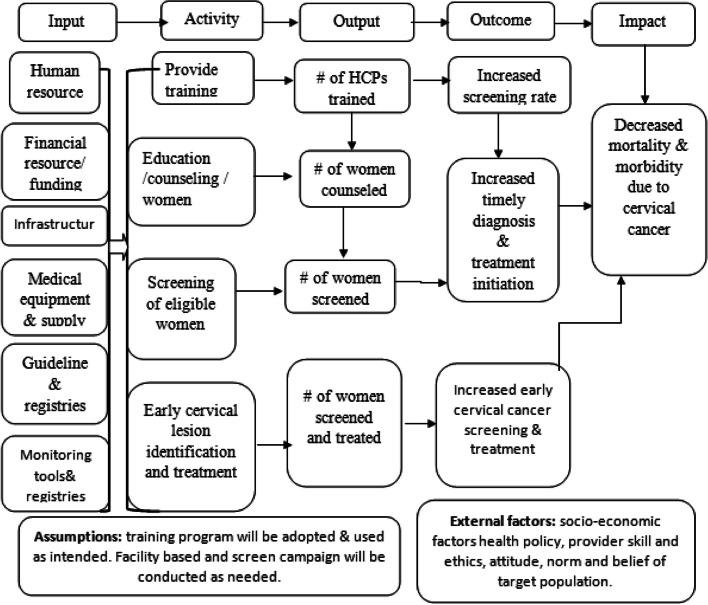
Adapted logic model for cervical cancer screening program implementation [13]

Logic model is a diagrammatic expression of program components, assumptions, external factors and their relation in the creation desired results**.** It indicates the sensible connection among input, activity, output, outcome and impact with arrows to indicate causal relationships between them (Fig. 1).

*Statement of problem*: cervical cancer burden is high in Ethiopia that an estimated 7095 new cases and 4732 women deaths from the disease annually [7].

*Goal*: To contribute for the reduction of morbidity and mortality related to cervical cancer in Gondar city administration (Fig. 1).

## Evaluation questions and objectives

### Evaluation questions

- Are the required resources available to provide cervical cancer screening with VIA?
- Do health care workers provide cervical cancer screening, treatment and follow-up service based on the national screening guidelines?
- What is the level of client satisfaction and associated factors with cervical cancer screening services?

### Objectives of evaluation

#### General objective

To evaluate visual inspection with acetic acid cervical cancer screening program implementation in Gondar city administration health centers, Northwest, Ethiopia.

#### Specific objectives

➢ To assess the availability of resources required to provide cervical cancer screening with VIA in Gondar city administration public health facilities, North West Ethiopia, 2021.
➢ To evaluate compliance of healthcare workers to the national cervical screening procedure guidelines during service delivery in Gondar city administration public health facilities, Northwest Ethiopia, 2021.
➢ To determine the level of client satisfaction with cervical cancer screening services in Gondar city administration public health facilities, North West Ethiopia, 2021.
➢ To identify factors associated with client satisfaction in Gondar city administration public health facilities, North West Ethiopia, 2021.

## Evaluation methods and materials

### Evaluation area and period

This evaluation was conducted from March 01–30 2021 in Gondar city administration public health facilities. Gondar city is found in the Amhara National Regional State, Central Gondar Zone which is 727 km from Addis Ababa, the capital of Ethiopia. The city administration had nine public health facilities (eight health centers and one teaching and specialized hospital) that has being served for over 700,000 people. It had also more than 20 private health facilities including primary hospitals, specialty clinics and primary clinics. In the eight health facilities there are fourteen trained health care providers and a total of 391 clients were screened for cervical cancer in the last quarter. Cervical screening and early treatment service is given in all public health facilities.

### Evaluation design and approach

A single case study design with a concurrent mixed-method was used to evaluate cervical cancer screening with VIA. Formative approach was applied to identify the gaps that hinder the achievement of desired results. This formative evaluation helps to identify potential area of concern, area of improvement, and generate data on the need for program improvement. The evaluation wants to answer how and why questions and needs to understand the availability of resources, compliance of HCWs and satisfaction of clients. The qualitative findings were used to triangulate and support the quantitative results.

### Evaluation dimensions

This evaluation focused on understanding and describing program’s implementation theory components and some immediate outcomes like client satisfaction. The availability and acceptability dimensions from access framework and compliance dimension from fidelity frame work were used to evaluate and judge cervical screening with VIA implementation.

### Population and sampling

#### Source population

The source populations for this evaluation were all reproductive age group females who live in Gondar city and all HCWs in Gondar city administration public health facilities.

#### Study population

The study populations were all reproductive age females received cervical screening services and all HCWs providing cervical cancer screening service at Gondar City Administration health centers during data collection period.

### Sample size determination

#### For exit interview

The sample size for exit interview was calculated by using single population proportion formula by considering 95% confidence interval, 5% margin of error and population proportion 76.4 (from a study conducted in Addis Ababa) [14]).

$$\begin{array}{c}\mathrm{n}={\left(\frac{Za}{2}\right)}^{2}*p*\left(1-p\right)/{d}^{2}\\ \mathrm{n}=({1.96)}^{2}*0.76*(1-0.76)/{(0.05)}^{2}\\ \mathrm{n }=281\end{array}$$

and assumed non-response rate is 10%, then the final sample size becomes 310.

#### For qualitative data

To assess HCPs compliance to the national standard, a total of 30 non-participatory client-provider interaction observations have been done. The observers had no interference with the procedure even the HCPs come across a malpractice.

For resource availability, a structured checklist which was filled out by observing the existences of each item for eight health centers was conducted.

The number of key informant interviews were determined by data saturation [15]. Based on this principle a total of 14 key informant interviews (eight focal persons and six administrative) were included. The key informant guide included all thematic areas and saturation was considered in these themes.

#### Sampling procedure for quantitative

**Fig. 2:**
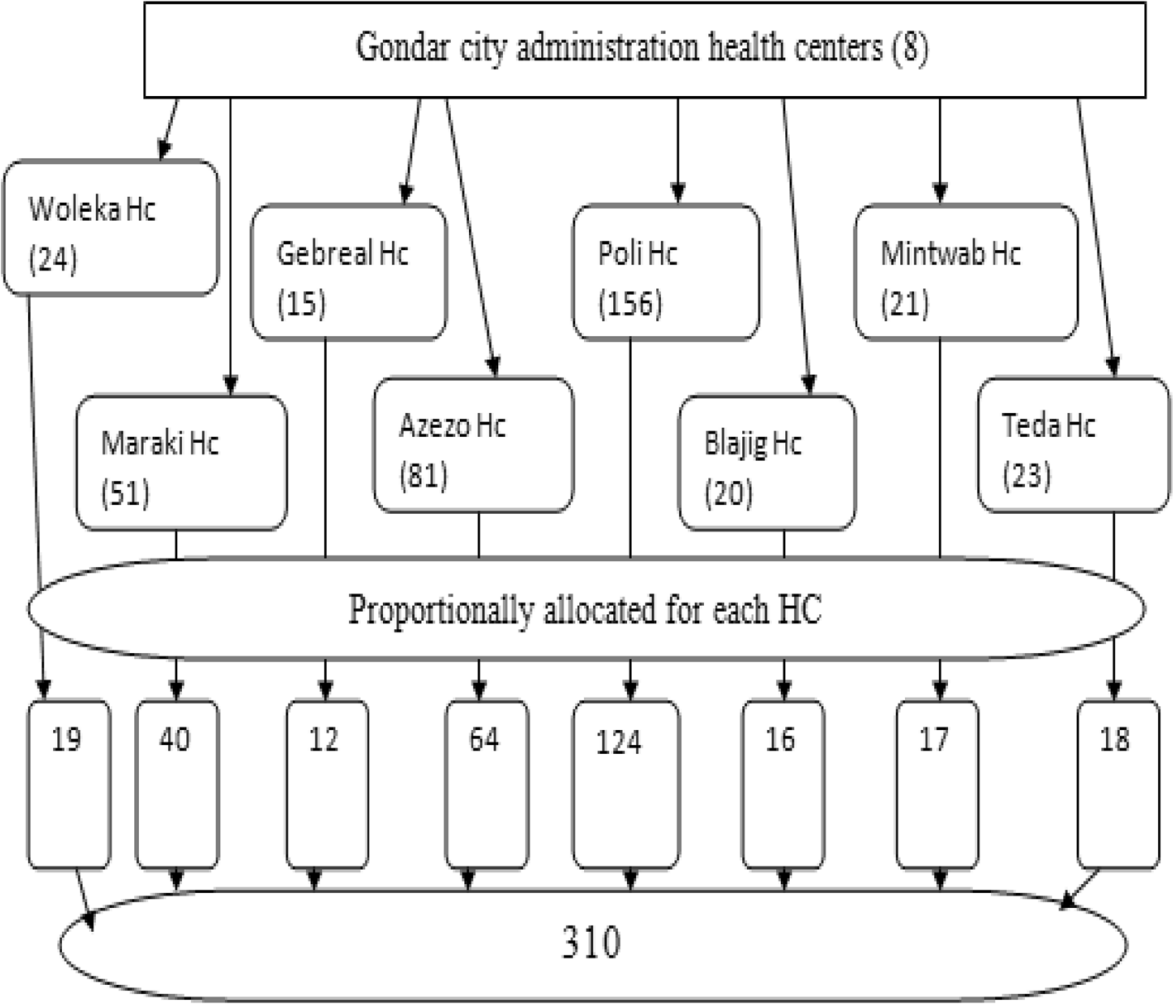
Sampling procedure for client exit interview to evaluate Cx Ca screening program in Gondar city administration Hcs in 2021

The calculated sample size was proportionally allocated for each health centers based on last quarter client flow reports. Then a consecutive sampling technique was employed to select study participants in each health center to measure their satisfaction (Fig. 2).

#### Inclusion and exclusion criteria

All females getting cervical cancer screening service in Gondar administration health centers during data collection period and all health care providers working in the cervical cancer screening program were included in this evaluation.

Health care providers providing cervical screening service for less than six months were excluded from the study.

#### Data collection tools and field work

Resources availability assessment checklists and key informant interview questionnaires were adapted from previously published articles [16–18]. The compliance of health care providers was assessed by check lists which were adapted from literatures [11, 19, 20]. These tools were translated to Amharic (local language) for easily understanding. A senior research expert was involved during the tool adaptation and translation (mentioned in the acknowledgment). Exit interview questionnaires were developed and pretested at the non-selected health center, Maksegnit health center to check tool validity.

Two diploma nurse data collectors and one health officer supervisor were recruited and two days training was given. The two nurse conducted exit interview with close supervision of the health officer. Key informant interview and client-provider interaction observation were conducted by the principal evaluator. The collected data were checked for completeness and consistency before coding and entering in to software.

#### Data management and analysis

The quantitative data were entered into EPI data version 4.6 and exported to SPSS version 20 software for analysis. The qualitative data were transcribed, translated and themes were formed manually. The description of the program implementation in terms of resources availability, health care providers’ compliance and client satisfaction were presented by using tables and narrations. For regression analysis; variables in binary logistic regression with 95% CI, 5% margin of error and *p*-value < 0.025 were taken to multilevel logistic regression. Finally variables with *p*-value ≤ 0.05 in the multivariable logistic regression were declared as associated variables with client satisfaction.

#### Matrix of analysis and judgment

The matrix of analysis and judgment were developed with full involvement of stakeholders during evaluability assessment phase. The weight of dimensions and the respective indicators were given depending on their level of relevance to the program. The sum of all dimensions was attributed to the service's implementation status. Therefore, by convention the stakeholders gave the weight of resources availability 35%, health care providers’ compliance 30% and client satisfaction 35% (Table 2).

**Table 2:** Judgment matrix for the overall implementation of cervical cancer screening program in Gondar city administration health centers, 2021 [21]

Dimensions	Weight given (%)	No. of indicators used	Achievement (%)	Judgment
Resource availability	35			≥ 85—v. good 70–84—good 60–69—fair < 60–poor
HCP compliance	30			
Client satisfaction	35			
Over all Cx Ca screening program implementation	100			

## Results

### Availability

A total of eight health centers were observed to assess the availability of resources to deliver cervical cancer screening in Gondar city administration. Within the eight health centers eight midwives and six nurses were trained about cervical cancer screening and treatment and majority of them were females 11(78.6%).

From the key informant interview the majority of participants stated that there is a lack of trained HCPs at health centers.

*“I am the only trained health care provider working at cervical cancer screening with VIA. Because of shortage of health care providers in the health center, I am responsible to work in emergency department, dressing and injection besides in the cervical screening service*. [27 yrs. male BSc nurse]

Only five health centers had separate examination rooms and two health centers had 24 h electrical powers with back-up generator.

The majority of KII said that several requirements are not met to deliver quality cervical cancer screening.

*“Our health center has no separate room for cervical cancer screening. Giving cervical screening counseling and doing procedures within the outpatient room and maternity ward is difficult.” *[32 yrs. female BSc midwife]

There is no stopwatch in all health centers of the city. Only three health centers have gooseneck lamp or torch light. There are 12 speculums in each health center. But only three health centers had goose neck and none of them had stop watch (Table 3).

**Table 3:** Avaliability of resources required for cervical cancer screening with VIA implementation in Gondar city administration health centers, 2021

S. No		Available in HCs (during data collection time		Percent	
Health care workers		No. of HCWs	Trained (no.)	% of available HCWs	Trained (%)
1	Health officer	20	0	83	0
2	Nurse	86	6	98	7
3	Midwife	27	8	48	29.6
Infrastructures					
1	Separate examination room	5		63	
2	24 h electrical power with backup	2		25	
3	Hand washing area (sink with running water)	4		50	
4	Washroom/bathroom for client use	0		0	
5	Latrine rooms for patients	8		100	
6	Space for confidential counseling	8		100	
7	Shelf with separate room for instruments	1		13	
8	Specula (medium and large)	8		100	
9	Ring/ sponge-holding forceps	8		100	
10	Kidney dishes	8		100	
11	Rubber sheet	8		100	
12	Gynecological examination table covered by clean paper/cloth	8		100	
13	Gooseneck lamp/torchlight	3		38	
14	Instrument trays / trolleys	6		75	
15	Stopwatch	0		0	
16	Privacy screens	3		38	

From the key informant interview the majority of KII revealed that there is shortage of procedure materials.

*“Most of the program materials are not available in the market and supply agencies. Even though NGOs like family guidance association provides materials like speculum and gooseneck, it is not enough for all clients.” *[40 yrs. Male BSc laboratory]

During the data collection period, 3–5% acetic acid was available in100%. But only three health centers (38%) have national guidelines (Table 4).

**Table 4:** Avaliability of consumable resources and documents required for cervical cancer screening with VIA implementation in Gondar city administration health centers, 2021

S. no	Materials	Available during data collection		Available in the last 3 months	
		Number of HCs	Percent	Number of HCs	percent
1	Sterile examination glove	8	100	8	100
2	3–5% acetic acid	8	100	6	75
3	Small cotton swabs	7	88	7	88
4	Non-sterile gauze roll	7	100	8	100
5	sodium hypochlorite to make 0.5% solution	8	100	8	100

The majority of key informant interviewer response revealed that most of the consumable supplies were available except acetic acid.

“*There is shortage of acetic acid and due to that the program service were interrupted for the previous three days. Despite we bought the material from private market, the service is given for clients freely. This makes the sustainability of the program is being difficult.” *[29 yrs. male health officer]

### Availability dimension summary results

Availability of required resources for cervical cancer screening with VIA implementation was 52.3%. Based on the pre-setting judgment point this result needs major improvement (Table 5).

**Table 5:** Summary of availability dimension findings in evaluation of cervical cancer screening program in Gondar city administration health centers, 2021

S.№	Indicator	Expected	Observed	weight	Achieved in %	Judgment parameter	Judgment parameter criteria
1	Number of trained HCPs	18	14	6	78.2	Good	≥ 85% = V. good 70–84 = Good 60–69 = fair < 60 = poor
2	The proportion of health centers that have at least one national cervical screening guideline	1	0.38	1.5	38	Poor	
3	The proportion of health centers that had cervical cancer screening recording and reporting forms	1	0.88	2	88	V. good	
4	The proportion of health centers with separate examination rooms	1	0.63	4.5	63	Fair	
5	The proportion of health centers had hand washing area (sink with running water) in the procedure room	1	0.5	2	50	Poor	
6	The proportion of health centers had bathroom for clients	1	0	2	0	Poor	
7	The proportion of health centers had shelf with separate room for instruments	1	0.13	1.5	13	Poor	
8	The proportion of health centers had adequate number of speculum	1	0.63	3	63	Fair	
9	The proportion of health centers had adequate number of kidney dish	1	0	1	0	Poor	
10	The proportion of health centers have rubber sheet that meet the standard number	1	0.25	1	25	Poor	
11	The proportion of health centers had gooseneck lamp / torchlight/	1	0.38	2	38	Poor	
12	The proportion of health centers had stopwatch	1	0	2	0	Poor	
13	The proportion of health centers used a screen to protect client privacy	1	0.38	1.5	38	Poor	
14	The proportion of health centers had consumable supplies	1	0.75	5	3.75	Good	
Total				35	18.29	poor	

NB: proportion is b/n 0 and 1, Achievement = (observed/expected)*100

## Compliance

A total of 30 non-participatory observations were conducted over eight HCPs during their counseling and performing the cervical cancer screening procedure. The number of observations was determined by saturation point per a single HCP. On average seven observations were conducted per individual HCP and the first three observations were discarded to reduce the Hawthorne effect.

Most of the HCPs dressed based on the dressed code (93%) and give greeting for clients with respect (80%). But only 37% of HCPs ask a woman for verbal consent and very few (16.7%) of them wash their hands with water and soap after procedure. HCPs used a cotton swab to remove any discharge from the cervix before using acetic acid was found to be 18 (60%). HCPs who saw the cervix for at least one minute following the application of acetic acid were 22(73%). After the procedure, 9 (30%) HCPs used fresh cotton balls to remove acetic acid from the cervix and vagina (Table 6).

**Table 6:** Observation of client-providers interaction during cervical cancer screening in Gondar city adminesration public health facilities, 2021; *n* = 30 observations

S/No	Activities	Expected	Observed	Weight	Achievement in %	Judgment
1	Dress based on dressing code of ethics	30	28	2	93	Good
2	Give greeting for clients with respect	30	24	1	80	Good
3	Apply good counseling skills (asking open-ended questions)	30	20	2	67	Fair
4	Assure the woman that her information is confidential during counseling	30	5	1	16.7	Poor
5	Explain how VIA test prevent cervical cancer	30	18	2	60	Fair
6	Make clear how the VIA test is done	30	16	1	53	Poor
7	Ask a woman for verbal consent	30	11	1	37	Poor
8	Wash hands thoroughly with soap & water before and after procedure	30	0	2	0	Poor
9	Properly wear on examination gloves	30	23	1	77	Good
10	Use drapes to cover woman during an examination (privacy)	30	0	1	0	
11	Insert speculum and adjust light source	30	30	2	100	Good
12	Use cotton swab to remove discharge, blood or mucus from cervix	30	18	1	60	Fair
13	Observe the cervix at least one minute after acetic acid application	30	23	1	77	Good
14	Remove acetic acid from cervix & vagina using fresh cotton balls	30	9	2	30	Poor
15	Remove gloves by turning inside out	30	23	3	77	Good
16	Wash hands with water and soap after procedure	30	5	2	16.7	Poor
17	Tell (inform) to the woman about her result	30	30	1	100	Good
18	Advise a woman to return for a repeat test after three or five years if she is negative	30	25	1.4	83	Good
19	Explain to woman the necessity of further investigations if, she is positive	30	5	0.6	16.7	Poor
20	Record VIA result in the register	30	30	1	100	Good
21	Note down decision made on VIA record	30	30	1	100	Good
Total						

From the key informant interview the majority of the participants stated that there is no guideline in their health center.

*“In our health center only two midwives covered cervical cancer screening, ANC, PNC and delivery services. To perform the procedure according to the standard it needs more time. Cleaning and sterilizing the procedure materials is another burden*.*” *[32 yrs. female BSc midwife]

## Satisfaction

### Sociodemographic characteristics

A total of 310 participants with a response rate of 98.7% were included in the exit interview. The participants’ mean age was 35.8 (SD ± 5.3) years, with a range of 19 to 49 years. Furthermore, the majority of participants were orthodox followers 269 (87.9%), urban dwellers 242 (79.1%) and more than half of the participants were married160 (52.3%) (Table 7).

**Table 7:** Socio-demographic characteristic of participants in the evaluation of cervical cancer screening program in Gondar city administration public health facilities, 2021

Variable		Frequency(*n* = 306) (*n* =	Percent
Age in years	19–30	19	6.2
	30–34	94	30.7
	35–39	121	39.5
	40–44	55	18.0
	45–49	17	5.6
Religion	Orthodox	269	87.9
	Muslim	29	9.5
	Protestant	4	1.3
	Others*	4	1.3
Residence	Rural	64	20.9
	Urban	242	9.1
Travelling time to nearest HF	< 30 min	181	59.2
	≥ 30 min	125	40.8
Marital status	Single	29	9.5
	Married	160	52.3
	Divorced	89	29.1
	Widowed	22	7.2
	Separated	6	2.0
Educational status	Unable to write and read	82	26.8
	Write and read only	10	3.3
	Primary (1–8)	115	37.6
	Secondary (9–12)	65	21.2
	College and above	34	11.1
Occupational status	Farmer	28	9.2
	Housewife	95	31.0
	Government employee	27	8.8
	Merchant	69	22.2
	Prostitution	18	5.9
	Others**	69	22.5

Keys: * = Jewish, ** = daily labor, mill work, private agency, private work (beauty salon, coffee), charitable agency, restaurant, jobless, student

The majority of participants 268 (86.5%) had heard about cervical cancer screening program previously. The sources of the information were HCPs (58.9%), radio/TV (21%), relative or neighbors (10%) and newspaper (1%).

### Reproductive health related characteristic of participants

In this evaluation majority of the participants 265 (86.6%) had one or more history of giving birth. The evaluation result also showed that 59 (19.3%) of the participants have lifetime history of STI and 301 (98.4) had tested for HIV (Table 8).

**Table 8:** Reproductive characteristic of the participants in the evaluation of cervical cancer screening program in Gondar city administration public health facilities, 2021

**Reproductive history variable**		Frequency	Percent
Give birth previously	No	41	13.4
	Yes	265	86.6
No. of birth	1–2	136	44.4
	3–4	98	32.0
	≥ 5	31	10.1
Lifetime history of STI	No	247	80.7
	Yes	59	19.3
STI diagnosed clients get cervical cancer screening service	No	4	6.8
	Yes	55	93.2
Age at first intercourse	No remember	44	14.4
	13–15 years	62	20.3
	16–18 years	113	36.9
	19–24 years	81	26.5
	≥ 25 years	6	2
Lifetime sexual partner	No	15	4.9
	1	139	45.4
	2	105	34.3
	More than 2	47	15.4
HIV test performed	No	5	1.6
	Yes	301	98.4
HIV result (300)	Non-reactive	197	65.6
	Reactive	103	34.1
	Not receive the result	1	0.3

### Client satisfaction

Client satisfaction was measured by using a five point likert scale questions. For regression analysis; clients who answered above the mean value were considered as satisfied and below the mean value as not satisfied.

Nearly half of the participants (47.7%) are very satisfied with the amount of time they spent with HCPs. More than half of the participants (52.6%) very satisfied with the overall service and recommend the services to their family and neighbors (50.7%) (Table 9).

**Table 9:** Client satisfaction with cervical cancer screening program services in Gondar city public health facilities, 2021

**Satisfaction item**	Very dissatisfied no. (%)	Dissatisfied no. (%)	Neutral no. (%)	Satisfied no. (%)	Very satisfied no. (%)
HCPs respect and approach me friendly	5(1.6)	15(4.9)	1(0.3)	118(38.6)	167(54.6)
The distance from home to health facility is not far	20(6.5)	36(11.8)	2(0.7)	115(37.6)	133(43.5)
There was appropriate chairs in the waiting area	28(9.2)	73(23.9)	4(1.3)	153(50.0)	48(15.7)
Waiting time to get screening service was appropriate	11(3.6)	59(19.3)	7(2.3)	134(43.8)	95(31)
The opening time of health center was appropriate	23(7.5)	87(28.4)	34(11.1)	116(37.9)	46(15.0)
The time spent with HCPs was appropriate	3(1.0)	12(3.9)	3(1.0)	142(46.4)	146(47.7)
HCPs explained what is cervical cancer and the procedure	18(5.9)	33(10.8)	7(2.3)	131(42.8)	117(38.2)
HCPs are involved me in decision about services	32(10.5)	49(16.0)	4(1.3)	138(45.1)	83(27.1)
I feel HCPs keep the confidentiality of my information	5(1.6)	11(3.6)	20(6.5)	126(41.2)	144(47.1)
HCPs keep my privacy during procedure	8(2.6)	33(10.8)	13(4.2)	98(32.0)	154(50.3)
HCPs gave good care of me today	5(1.6)	12(3.9)	2(0.7)	136(44.4)	151(49.3)
I feel good during procedure	26(8.5)	84(27.5)	4(1.3)	138(45.1)	54(17.6)
HCPs listened me intently	3(1.8)	7(2.3)	2(0.7)	176(57.5)	118(38.6)
HCPs answered all my questions	5(1.6)	19(6.2)	31(10.0)	167(54.6)	84(27.5)
In my opinion HCPs are qualified	0	13(4.2)	22(7.2)	142(46.4)	129(42.2)
The Examination room is clean	31(10.1)	60(19.6)	5(1.6)	149(48.7)	61(19.9)
Bathrooms and toilets are kept clean	64(20.9)	54(17.6)	94(30.7)	68(22.2)	26(8.5)
I recommend that my relatives and neighbors to get the service	8(2.6)	18(5.9)	7(2.3)	155(50.7)	118(38.6)
Overall satisfaction with the service received?	4(1.3)	17(5.6)	4(1.3)	120(39.2)	161(52.6)

### Factor associated with client satisfaction

In this evaluation residence of clients, travel distance to nearest health facility, educational status, having information on cervical cancer screening, history of STI, number of lifetime sexual partner and HIV status were candidates for the multi variable logistic regression. In the multi variable logistic regression women’s educational status, having previous information on cervical cancer screening and number of lifetime sexual partners were associated variables with client satisfaction.

Accordingly, clients who had secondary and above educational status were 3.58 times more satisfied than who had no formal education (AOR = 3.58, 95%CI (1.83–7.02), *p*-value = 0.00).

Clients who had previous information regarding cervical cancer screening were 2.24 times more satisfied than those who did not have information (AOR = 2.24, 95 percent CI (1.08–5.14), *p*-value = 0.02).

Clients who had three and more lifetime sexual partners were 4.62 times satisfied with cervical cancer screening program services than those who had never (AOR = 4.62, 95 percent CI (1.64–11.62), *p* = 0.03) (Table 10).

**Table 10:** Bi-variable and multi-variable logistic regression analysis for clients’ satisfaction on cervical cancer screening program services in Gondar city administration, 2021

Variables		Category		COR (95% CI)	AOR (95% CI)
		Satisfied	Dissatisfied		
Residence	Rural	29	35	1.00	1.00
	Urban	145	97	1.80(1.04–3.14)	.91(.48–1.72)
Travel time to nearest HF	< 30 min	112	69	1.65(1.04–2.62)	.59(.34–1.06)
	≥ 30 min	62	63	1.00	1.00
Educational status	No formal education	36	56	1.00	1.00
	Primary	66	49	2.09(1.20.3.66)	1.81(.98–3.35)
	Secondary and above	72	27	4.15(2.26–7.63)	3.58(1.83–7.02) *
Having information on cervical cancer screening	Yes	153	96	2.73(1.20–4.66) *	2.24(1.08–5.14) *
	No	21	36	1.00	1.00
History of STI	Yes	42	17	2.15(1.16–3.99)	.74(.36–1.52)
	No	132	115	1.00	1.00
Life time sexual partner	< 3	133	126	1.00	1.00
	≥ 3	41	6	6.4 (2.66–15.77)	4.62(1.64–11.62) *
HIV status	Non-reactive	100	97	1.00	1.00
	Reactive	68	35	.53(.32-.87)	1.55(.85–2.82)

Key: * showed significant association at *p*-value ≤ 0.05 on multi variable logistic regression model

The overall satisfaction of clients with cervical cancer screening services was found 77.1%, which is good and partially implemented. The overall cervical cancer screening program implementation in terms of availability, compliance and acceptability as satisfaction was 64.5% and judged as fairly implemented (Table 11).

**Table 11:** Matrix of analysis & judgment of cervical cancer screening program in Gondar city adminestration public health facilities, 2021

Dimensions	Expected %	Indicators	Weight	Score	Observed %	Judgment
Availability	100%	14	35	18.29	52.3	Poor
Compliance	100%	19	30	19.20	64.3	Fair-
Satisfaction	100%	20	35	27.00	77.1	Good
Overall program implementation	100%	53	100%	64.5	64.5	Fairly implemented

## Discussion

This research determines cervical cancer screening program implementation in terms of availability of required resources, compliance of HCPs to the standards and satisfaction of clients. Accordingly, cervical cancer screening program in Gondar city administration public health facilities were implemented fairly but needs further improvement. Almost half of the public health facilities had no required resources for cervical cancer screening and early treatment. Around two third of the HCPs implemented the procedure based on the standards and over three fourth of the clients were satisfied by the service they received.

In this evaluation, ten HCPs within the eight health centers, at least one per health facility were trained on cervical cancer screening program. This finding is congruent with the result in the Service Availability and Readiness Assessment (SARA) annual monitoring report [17]. Studies also recommended that all service providers in a program must maintain their knowledge and abilities by attending appropriate trainings, refreshment courses, and facility-level technical update meetings on a regular basis [11]. In this evaluation only two health centers had 24 h electrical powers with back-up generator. This finding was inconsistent with the study conducted by Addis Tesfa project that showed electrical power frequently was interrupted in all 14 sites [16]. The reason behind this variation might be that the project study was conducted on tertiary and secondary level health facilities [16]. Accordingly, only half of the health centers had hand washing area but water was frequently interrupted. The finding was consistent with the studies conducted by Addis Tesfa project [16]and WHO [22].

Our finding showed that on average 12 speculum, 13 forceps and 3 kidney dishes were available in each health center. The result was not in line with the standard requirement [23, 24]. Some of the required supplies like acetic acid, stop watch and gooseneck were insufficient. This is due to the materials were not available at the market with feasible cost.

According to this evaluation finding, almost two-third of HCPs did cervical cancer screening based on the national guidelines. The finding was judged as fair but needs further improvements.

The HCPs had counseled for two-third of the clients and this finding is lower than a study in Ghana [25]. In our evaluation finding HCPs respect for clients, applying acetic acid based on the standard and assurance of women for information confidentiality were also lower than the study findings in Ghana [25]. The reason may due to HCPs’ workload and training gaps in our evaluation site.

Even though the overall client satisfaction was judged as good, it needs improvement. In our evaluation finding almost all clients had previous information about the program on HCPs, radio or TV and from their families or neighbors. This result is supported by a study finding in Malawi [26], Addis Ababa [9] and Moroco [27]. In this evaluation almost all of the women would suggest the VIA test to their relatives and friends, which is consistent with study in Moroco, India and Malawi [26–28]. Only two-fifth of the clients complained about discomfort and pain during the VIA test procedure, which is a slight greater than a study finding in Moroco [27] and rural India [28]. This variation may be related to experience of HCPs and the use of in appropriate speculum size.

This evaluation showed that clients who had secondary and above educational status were more satisfied than who had no formal education. The finding is congruent with a study conducted among HIV positive women in Lagos, Nigerian [29], Dare Salaam [30] and in Addis Ababa [9].

Participants who had previous information regarding cervical cancer screening were more satisfied than those who did not have. This result also similar with the study conducted in Mozabique [31].

## Strength and limitation of the evaluation

This evaluation used both qualitative and quantitative methods with different data sources to get more accurate and detailed results.

Hawthorn effect during observation might have contributed to relatively high-performance scores. To reduce this effect we dropped the first three observations. Due to the feasibility issue we used only three dimensions. Judgment of the program implementation with only the three dimensions may not be fair and we recommended for other evaluators to include other dimensions. Besides, HCPs and institutions related factors were missed during our data collection.

## Conclusion

This evaluation found that cervical cancer screening by using VAI in Gondar city administration public health facilities was fairly implemented and needs improvement. Although half of the health facilities had VIA cervical cancer screening procedure rooms, they lack a hand washing area and sink. Most key informants complained that lack of trained HCPs, unavailability updated guidelines, and shortage of consumable materials were the challenges HCPs not to be compliant during the program implementation. According to this evaluation, most of the women who got cervical cancer screening service were satisfied. But participants’ satisfaction with the opening time of the health facilities, cleanliness of the bath room and toilet rooms was poor. Being educated and having information on cervical cancer screening previously were significantly associated with the acceptability of cervical cancer screening program.

In order to improve the program, FMoH and ARHB shall provide regular training and post-training supportive supervision to improve HCPs compliance with the standards. The city administration health office is better to strength the linkage with referral hospitals to improve VAI cervical cancer screening procedure performance. Equipment and resources such as speculum, forceps, torchlight, timer and acetic acid shall be availed and routinely supplied through pharmaceutical supply like EPSA. The health facilities’ heads shall avoid service interruption due to equipment and supply shortage, purchase the equipment and supply that are available in private suppliers.

## Data Availability

Data will be available upon reasonable request from the corresponding author.

## Abbreviations

EPSA: Ethiopia Pharmaceutical Supply Agency
FMOH: Federal Ministry of Health
HCP: Health Care Providers
HIV: Human Immune Virus
LMIC: Low-Middle Income Countries
RH: Reproductive Health
SSA: Sub-Saharan Africa
STI: Sexual Transmitted Infection
VIA: Visual Inspection with Acetic acid
WHO: World Health Organization
